# sIgE to peanut components does not accurately predict the severity of allergy in subjects suspected of peanut allergy

**DOI:** 10.1186/2045-7022-5-S3-P34

**Published:** 2015-03-30

**Authors:** Rob Klemans, Yolanda Meijer, Francine Van Erp, Cornelis Van der Ent, Carla Bruijnzeel-Koomen, Henny Otten, Edward Knol, Suzanne Pasmans, André Knulst

**Affiliations:** 1 University Medical Center Utrecht, Utrecht, The Netherlands

## Background

Many recent studies have shown the superior diagnostic accuracy of specific IgE (sIgE) to Ara h 2 compared to sIgE to peanut extract in diagnosing peanut allergy. The aim of this study was to analyze the association between severity and sensitization levels (by SPT, sIgE to peanut extract and peanut components Ara h 1, 2, 3 and 8).

## Methods

Peanut allergic adults (n=94) and children (n=100) with a suspected peanut allergy were included. sIgE levels were measured by ImmunoCAP (Uppsala, Sweden). In all subjects the adapted Mueller score was used with one additional category for no symptoms (0-5): 0; no symptoms/peanut tolerant, 1; oral allergy symptoms, 2; cutaneous/mucosal symptoms, 3; gastrointestinal symptoms, 4; respiratory symptoms, 5; cardiovascular symptoms. A severe reaction was defined as a Mueller score of 4 or 5. Univariate and multivariate logistic regression analysis was used to calculate odds ratio’s.

## Results

Children had significantly higher Mueller scores than adults (P=0.003) and were therefore analyzed separately. In children 12 patients had a severe allergy, in adults 15 patients. In the pediatric population, none of the predictors was significantly associated with a severe peanut allergy, since the 95% CI of the OR of all predictors included the value 1. In the adult population, sIgE to Ara h 2 (OR 1.03, 95% CI 1.01-1.06) and sIgE to peanut extract (OR 1.02, 95% CI 1.003-1.05) were significantly associated with a severe peanut allergy. A multivariable logistic regression analysis was not possible due to the low number of severely allergic patients. The AUC values of sIgE to Ara h 2 and sIgE to peanut extract in the adult population were 0.81 (95% CI 0.69-0.93) and 0.70 (95% CI 0.54-0.86), respectively (compared to 0.77 (0.64-0.89) and 0.69 (0.53-0.85) in the pediatric population).

## Conclusion

In children no predictor was significantly associated with severity of peanut allergy. In adults, specific IgE to Ara h 2 was a better predictor for severe peanut allergy than sIgE to peanut extract, but was still suboptimal for this purpose.

